# Antiretroviral adherence and virologic suppression in partnered and unpartnered HIV-positive individuals in southern Brazil

**DOI:** 10.1371/journal.pone.0212744

**Published:** 2019-02-27

**Authors:** Marineide Gonçalves de Melo, Ivana Varella, Pamina M. Gorbach, Eduardo Sprinz, Breno Santos, Tauí de Melo Rocha, Mariana Simon, Marcelo Almeida, Rita Lira, Maria Cristina Chaves, Zoe Baker, Tara Kerin, Karin Nielsen-Saines

**Affiliations:** 1 Hospital Nossa Senhora da Conceição, Porto Alegre, Brazil; 2 UCLA Fielding School of Public Health, Los Angeles, California, United States of America; 3 Universidade Federal do Rio Grande do Sul, Porto Alegre, Brazil; 4 David Geffen UCLA School of Medicine Department of Pediatrics, Los Angeles, California, United States of America; Côte de Nacre University Hospital, FRANCE

## Abstract

**Background:**

An undetectable serum HIV-1 load is key to effectiveness of antiretroviral (ARV) therapy, which depends on adherence to treatment. We evaluated factors possibly associated with ARV adherence and virologic response in HIV-infected heterosexual individuals.

**Methods:**

A cross-sectional study was conducted in 200 HIV-1 serodiscordant couples and 100 unpartnered individuals receiving ARV treatment at a tertiary hospital in southern Brazil. All subjects provided written informed consent, answered demographic/behavioral questionnaires through audio computer-assisted self-interviews (ACASI), and collected blood and vaginal samples for biological markers and assessment of sexually transmitted infections (STIs). HIV-negative partners were counseled and tested for HIV-1.

**Results:**

The study population mean age was 39.9 years, 53.6% were female, 62.5% were Caucasian, 52.6% had incomplete or complete elementary education, 63.1% resided in Porto Alegre. Demographic, behavioral and biological marker characteristics were similar between couples and single individuals. There was an association between adherence reported on ACASI and an undetectable serum viral load (P<0.0001). Logistic regression analysis demonstrated that single-tablet ARV-regimens were independently associated with adherence (OR = 2.3; 95CI%: 1.2–4.4; P = 0.011) after controlling for age, gender, education, marital status, personal income, ARV regimen, and median time of ARV use. A positive correlation between genital secretion PCR results and serum viral load was significant in the presence of STIs (r = 0.359; P = 0.017). Although HIV PCR detection in vaginal secretions was more frequent in women with detectable viremia (9/51, 17.6%), it was also present in 7 of 157 women with undetectable serum viral loads (4.5%), p = 0.005.

**Conclusions:**

ARV single tablet regimens are associated with adherence. Detectable HIV-1 may be present in the genital secretions of women with undetectable viremia which means there is potential for HIV transmission in adherent individuals with serologic suppression.

## Introduction

According to the WHO[1], approximately 1.7 million adults have recently become infected with HIV-1 and more than 90 percent of these infections are sexually transmitted, occurring in individuals of reproductive age. The city of Porto Alegre is the epicenter of the Brazilian HIV epidemic, with 74.0 cases per 100 thousand inhabitants, corresponding to twice the rate of the Rio Grande do Sul state and four times the Brazilian prevalence rate[2]. Antiretroviral (ARV) therapy has significantly improved the prognosis of patients infected with HIV-1 and has decreased the association with morbidity and mortality, besides reducing sexual transmission among serodiscordant couples[3]. Nevertheless, there are many challenges to the effective delivery of ARV, some of which are outlined in detail in the UNAIDS report of 2016[4].

Individuals in HIV serodiscordant relationships are at higher risk of acquiring HIV, and therefore are excellent candidates for prevention efforts. Through the landmark study *HIV Prevention Trial Network 052* (HPTN 052), the benefit of using ARV to prevent HIV-1 sexual transmission among serodiscordant heterosexual couples was clearly demonstrated[5]. Since the publication of this study, the World Health Organization (WHO) recommended the utilization of ARV as a method for prevention of HIV-1 transmission in this population[6]. Given the benefits demonstrated by HPTN 052, current research efforts focus on the effectiveness of antiretroviral treatment as a real-world prevention tool outside of the safety of a well-designed controlled clinical trial in an eligible patient population[7]. This is a particularly important point, because despite the current advances in prevention strategies, the number of new cases of HIV-1 transmission continues to increase as demonstrated by country statistics[1]. One of the main determinants of HIV-1 transmission in serodiscordant partners is adherence of the HIV-1 index partner to ARV[8].

With the increased availability of effective ARV treatment regimens, people living with HIV/AIDS have longer and healthier lives and maintain active sexual lives. Condom use is the main option for safe sex among HIV-serodiscordant couples, reducing the risk of sexual transmission by 80%[9]. However, condom use does not meet the sexual needs of all People Living with HIV/AIDS (PLVHA) especially women who experience inequalities in negotiating its use, or couples who wish to conceive, or for those who wish to increase sexual pleasure and intimacy. Another approach, which is the use of ARV to prevent sexual transmission has proven to be more successful. In fact, the success of ARV for prevention of sexual transmission of HIV-1 among heterosexual HIV serodiscordant couples was so great that, in 2008, the *Swiss Federal Commission* for HIV/AIDS established that HIV-positive heterosexual individuals in effective ARV use who had and undetectable plasma viral load for up to 6 months and had no STIs in this period could be considered non-infectious. This statement led to considerable debate on the possible risks of sexual disinhibition and risk compensation in this population which could counterbalance the benefits of ARV as a prevention strategy[7, 8, 10–14]. However, with the release of data from the HPTN 052 study, the evidence in support of the Swiss Declaration is undeniable today and there is a global movement[5, 7, 8] for treatment as prevention (TasP). In this setting, HIV-infected sexual partners with an undetectable viral load are considered to have zero risk of sexual transmission to their serodiscordant partners. TasP has fundamentally changed the focus on prevention of HIV sexual transmission from condom use to adherence to ARV use. Awareness that ARV prolongs life, improves life quality, and prevents HIV transmission encourages individuals to adhere to treatment promoting safer sexual practices[15–20].

Despite this newly acquired knowledge, it is clear that the biology of HIV-1 sexual transmission and the interaction with human sexual behavior are complex. Therefore it is necessary to evaluate these parameters in various regions of the world and within different contexts taking into account circulating viral subtypes, viral load of genital secretions, presence of STIs and behavioral characteristics. In addition, safe sex in HIV affected individuals today implies regular use of ARV in order to attain undetectable viral load and also condom use. Nevertheless, although serum viral load below detectable limits means suppression of HIV-1 and is the key to the efficacy of ARV use[14, 21], not all patients adhere to treatment[22–24].

In light of these findings, the main objective of the present study was to evaluate adherence to ARV among individuals in HIV serodiscordant relationships (who presumably would have more motivation and support to adhere to ARVs) and adherence among unpartnered individuals. We aimed to evaluate adherence across treatment regimens and also evaluate behavioral factors that may be associated with adherence and virologic suppression. Additionally, we evaluated the association between self-reports of adherence through audio computer-assisted self-interviews (ACASI) with biomarkers of adhesion including plasma-free and vaginal secretion viral load. We also evaluated factors that may impact HIV-1 detection in genital secretions, such as concurrent STIs, a known biological factor associated with HIV transmission.

## Material and methods

A cross-sectional evaluation was performed in 200 couples (400 individuals) who were in a stable relationship for more than three months, and were serodiscordant for HIV-1, and in 100 single HIV-1 seropositive individuals with no partners at the time of the study. All HIV seropositive subjects had ongoing medical care and were on ARVs for three months or more at a specialized HIV care service at Hospital Nossa Senhora da Conceição in Porto Alegre, Brazil. All study subjects provided written informed consent, answered a demographic and behavioral questionnaire via ACASI and provided blood samples for viral load, CD4, STI assessments and vaginal/ genital secretions for virus load if female. All HIV-negative partners were counseled and tested for HIV-1. The study was reviewed and approved by the ethics committee/ Institutional Review Boards (IRB) of Conceicao Hospital (CEP Hospital Conceicao) and by the UCLA IRB (UCLA MIRB1).

Occasional alcohol intake was defined as the use of alcohol 1 to 4 times a month; frequent use was classified as alcohol use greater or equal to 2 to 3 times a week. Other parameters evaluated were those related to demographic or behavioral factors in individuals who were partnered or unpartnered, and biological markers in HIV-seropositive patients. Adherence was measured as self-reported ARV use in the past month. Self-reports of good, very good and excellent adherence were classified as “high adherence”. Reports of very little, little and intermittent use of ARV medication in the last month were classified as “poor adherence”. An undetectable HIV-1 virus load was used as the main measure of good adherence and was quantified through the Real Time Polymerase Chain Reaction (PCR) method (Abott). The reference value for non-detection was < 40 copies/mL. The viral load of genital secretions was evaluated by Real Time PCR method using the COBAS kit (Roche). The result was classified as undetectable when less than 17 copies/mL.

For statistical analysis, the Mann-Whitney U test was used to evaluate the differences between continuous variables with asymmetric distribution and Yates Chi-Square test was used for categorical variables. Fisher’s exact test was also used in the analysis. Anova One Way test was used for comparison of means. The Spearman correlation coefficient was used to evaluate the correlation between viral loads from different compartments with an asymmetric distribution, that is, the correlation between serum viral load and genital virus load in women according to HIV-1 genotype and presence of sexually transmitted infections. The correlation between serum viral load and vaginal secretion virus load was performed by first evaluating patients with STIs and subsequently evaluating patients without STIs in a stratified manner. We used the Spearman correlation coefficient to determine correlation. Median values between the three groups of individuals (HIV+ index cases, HIV- partners and HIV+ unpartnered individuals) were compared using the Kruskal Wallis test (WinPepi Program). Variables tested in a bi-variate analysis with P value higher than 0.25 were not included in the multivariate model. A multivariable logistic regression analysis was used to verify possible independent associations between stable relationship and ARV adherence controlling for potentially confounding factors such as demographic, behavioral characteristics and ARV regimens, whether simple or multiple with different factors tested for collinearity. Data was processed and analyzed by the programs Win PEPI version 11.5 and IBM *Statistical package of the social sciences* (SPSS) version 22.0.

## Results

**Fig 1:**
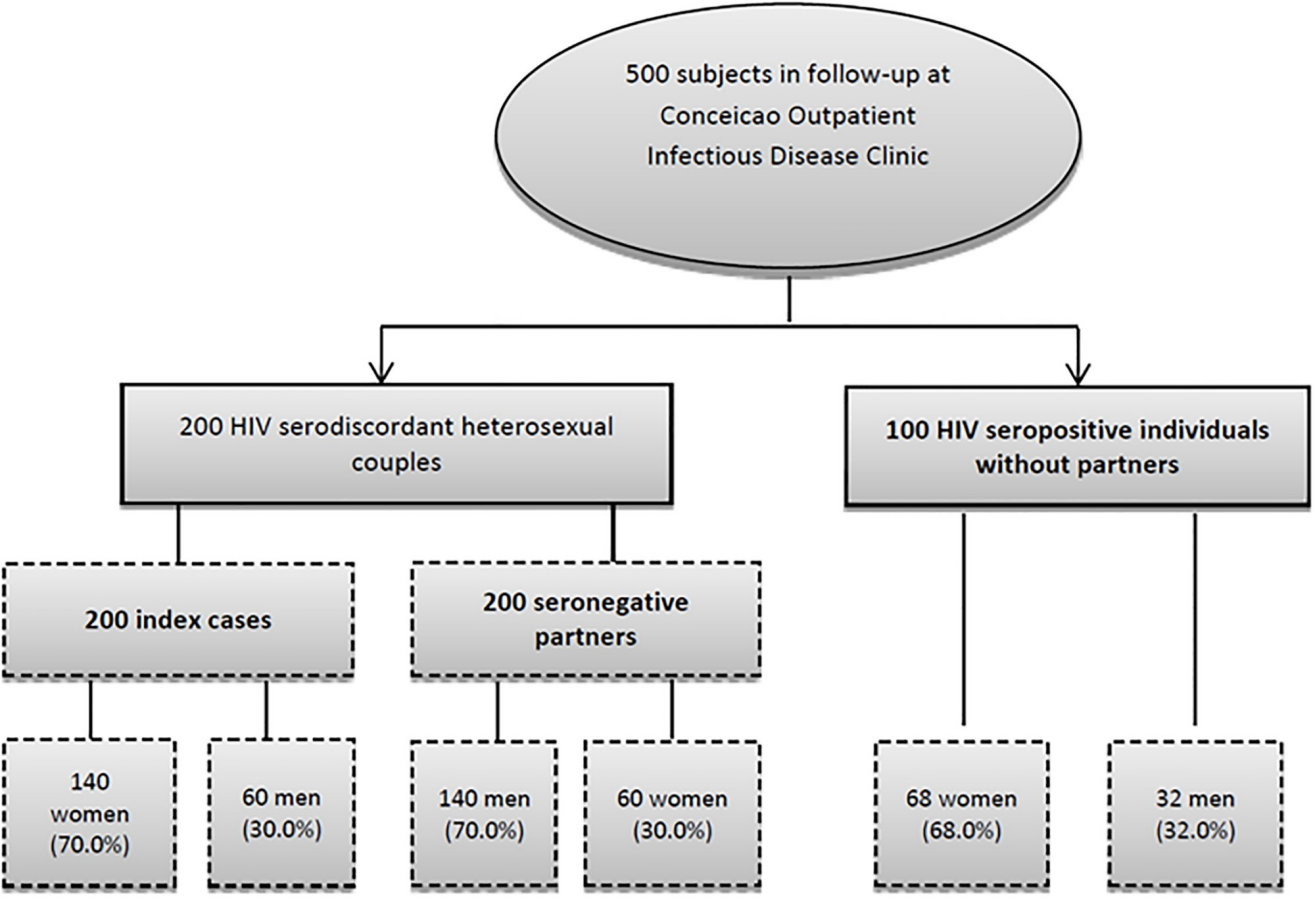
Diagram of study population.

The majority of the population studied (68.5%) consisted of HIV seropositive women (Fig 1). The mean subject age was 40 years. Most study participants lived in Porto Alegre (63.1%), were white (62.5%), female (53.6%) and had completed elementary school only (52.6%). The proportion of females and the mean age of unpartnered participants was higher than that of individuals in HIV serodiscordant relationships. Unpartnered individuals more frequently responded positively to having their own source of income, however the median monthly income was higher for couples. Demographic characteristics of the study population are shown in Table 1. Occasional alcohol intake was more frequently reported by couples. There were no reported differences between couples and unpartnered individuals regarding the use of illicit drugs, type of drugs or injectable use (Table 2).

**Table 1:** Demographic characteristics of the study population stratified by index cases, HIV uninfected partners and HIV infected unpartnered individuals (n = 500).

Characteristic	Total	Couples (+) HIV	Couples (-) HIV	Single individuals	P*
		N° (%)			
	n = 500	n = 200	n = 200	n = 100	
Age, years ^(1)^	39.9 ± 11.2 (18–73)	38.2 ± 9.8 (18–66)	39.9 ± 11.9 (18–73)	43.4 ± 11.5 (21–70)	<0.01
Age over 30 years	413 (82.6)	161 (80.5)	163 (81.5)	89 (89.0)	0.16
Female	268 (53.6)	140 (70,0)	60 (30.0)	68 (68,0)	<0.01
Race/ethnicity	n = 499	n = 200	n = 199	n = 100	
White	312 (62.5)	119 (59.5)	129 (64.8)	64 (64.0)	0.45
Brown	76 (15.2)	28 (14.0)	31 (15.6)	17 (17.0)	
Black	111 (22.2)	53 (26.5)	39 (19.6)	19 (19.0)	
Education	n = 498	n = 199	n = 199	n = 100	
EF ^(2)^	262 (52.6)	110 (55.3)	96 (48.2)	56 (56.0)	0.28
> EM ^(3)^	236 (47.4)	89 (44.7)	103 (51.8)	44 (44.0)	
Origin	n = 426	n = 173	n = 173	n = 80	
Porto Alegre	269 (63.1)	108 (62.4)	108 (62.4)	53 (66.3)	0.82
Other cities	157 (36.9)	65(37.6)	65(37.6)	27 (33.8)	
Presence of Personal Income (n = 500)	n = 500 382 (76.4)	n = 200 142 (71.0)	n = 200 156 (78.0)	n = 100 84 (84.0)	0.03
	n = 389	n = 145	n = 160	n = 84	
Monthly Family income, in reais^(4)^ (range)	1,500.00 (0–40,000.00)	1,700.00 (0–22,000.00)	1,600.00 (77–40,000.00)	1,000.00 (256.00–10,000.00)	<0.01
Q1,Q3	1,000.00; 2,500.00	1,000.00; 3,000.00	1,000.00; 2,950.00	788.00; 2,000.00	
Number of biological children	n = 500	n = 200	n = 200	n = 100	
No children	96 (19.2)	31 (15.5)	39 (19.5)	26 (26.0)	0.07
1 to 2	242 (48.4)	92 (46.0)	104 (52.0)	46 (46.0)	
3 or more	162 (32.4)	77 (38.5)	57 (28.5)	28 (28.0)	

* The 3 independent groups (HIV+ individual in couples, HIV-individual in couples, and unpartnered HIV+ individuals) were compared using the following tests: Pearson’s Chi square test for comparison of frequency distributions; ANOVA One Way test for comparison of means; Non-parametric Kruskal-Wallis test for comparison of median family income.

^(1)^ Results expressed in mean ± standard deviation (range);

^(2)^EF = incomplete or complete elementary school;

^(3)^EM = incomplete or complete high school;

^(4)^Results expressed in median (range); Q1, Q3 (interquartile 25 and 75).

**Table 2:** Characteristics of the study population as to alcohol or drug consumption as reported in ACASI (n = 500).

Characteristic	Total	Couples	Unpartnered	P*
		N° (%)		
	n = 500	n = 200	n = 100	
Report of alcohol use	229 (45.8)	199 (49.8)	30 (30.0)	<0.0001
Frequency of alcohol use				
Never^(1)^	271 (54.2)	201 (50.2)	70 (70.0)	-
Occasionally	186 (37.2)	164 (41.0)	22 (22.0)	<0.0001
Frequently	43 (8.6)	35 (8.8)	8 (8.0)	0.348
Use of illicit drugs	57(11.4)	42 (10.5)	15 (15.0)	0.206
	n = 57	n = 42	n = 15	
Use of more than one illicit drug	16 (28.1)	3 (31.0)	3 (20.0)	0.422
Types of illicit drugs				
Marijuana	25 (43.9)	18 (42.9)	7 (46.7)	0.800
Cocaine	19 (33.3)	12 (28.6)	7 (46.7)	0.206
Heroin	2 (3.5)	1 (2.4)	1 (6.7)	0.461
Amphetamine	2 (3.5)	1 (2.4)	1 (6.7)	0.461
IDU^#^	11 (19.3)	7 (16.7)	4 (26.7)	0.404

* P-value obtained through the Chi-square test with Yates correction.

^(1)^Reference Category.

^#^ IDU = injecting drug use.

Among all 500 participants, 20% had been recently treated for STIs (100 cases). This finding was more frequently observed in unpartnered individuals than in individuals in relationships (32.0% vs. 17.0%; P = 0.001). Unpartnered individuals more frequently had a diagnosis of hepatitis (22.0% vs. 6.5%; P<0.0001), while syphilis cases were more frequent among couples (17.3% vs. 7.0%; P = 0.008). When only STIs likely to cause genital lesions were considered, we identified 69 cases (23%) in the 300 HIV seropositive individuals in the study population; 44 STI cases occurred in HIV seropositive women (63.8%) and 25 in seropositive men (36.2%), P = 0.254. Among uninfected partners the frequency of STIs was 14.5% (P = 0.019).

**Fig 2:**
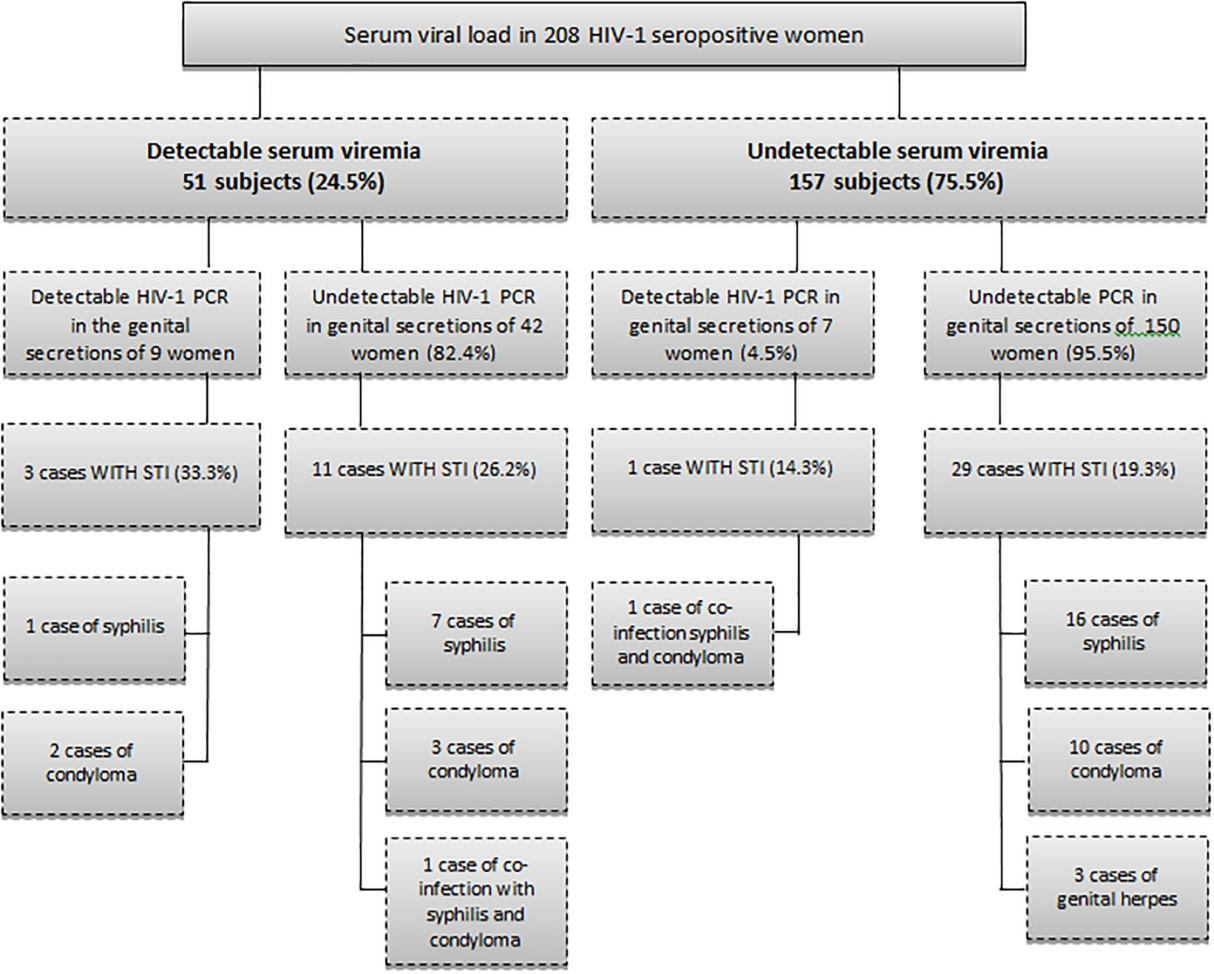
Viral load in serum and genital secretions of HIV-infected women in the study population. Greater chance of PCR detection in genital secretions of women with detectable serum viremia as compared to women with an undetectable serum viral load.

Among 300 HIV-infected subjects 20.3% had CD4 levels below 350 cells/μl and 22.7% a detectable serum viral load, without significant differences between index cases in couples and unpartnered individuals as seen in Table 3. However, the proportion of women with a detectable viral load in genital secretions was higher in unpartnered women than in women in stable relationships (13.2% vs. 5.0%; P = 0.037). The median viral load in genital secretions was also higher in unpartnered women as seen in Table 3. Among 208 HIV-positive women, detectable viremia was present in 51 subjects (24.5%) and genital viremia was observed in 16 (7.7%). There was a greater chance of PCR detection in genital secretions of women who had detectable serum viremia when compared to women with an undetectable serum viral load (17.6% vs. 4.5%; OR = 4.59; 95%CI: 1.41–15.31; P = 0.005). Among 157 women with an undetectable serum viral load, 7 cases of PCR-detectable HIV in vaginal secretions (4.5%) were identified (Fig 2).

**Table 3:** Results of biological markers, CD4 cell counts, serum virus load and virus load of vaginal secretions in HIV-seropositive individuals (index case in couple and unpartnered individuals) (n = 300).

	Total	Index**	Unpartnered	P*
Biological markers	n = 300	n = 200	n = 100	
		N° (%)		
CD4, cells/μl				
Below 350	61 (20.3)	39 (19.5)	22 (22.0)	0.613
350 or more	239 (79.7)	161 (80.5)	78 (78.0)	-
Serum viral load				
Detectable	68 (22.7)	44 (22.0)	24 (24.0)	0.697
Undetectable	232 (77.3)	156 (78.0)	76 (76.0)	-
Serum viral load in women	n = 208	n = 140	n = 68	
Detectable	51 (24.5)	35 (25.0)	16 (23.5)	0.818
Undetectable	157 (75.5)	105 (75.0)	52 (76.5)	
Viral load of genital secretions				
Detectable	16 (7.7)	7 (5.0)	9 (13.2)	0.037
Undetectable	192 (92.3)	133 (95.0)	59 (86.8)	-
Median values and interquartile ranges:	Total	Index	Unpartnered	
	n = 300	n = 200	n = 100	
CD4, cells/μl (range)	581.5 (7–2,657)	602.5 (41–1,658)	553.0 (7–2,657)	0.385
Q1;Q3	376.5; 807.5	389.5; 807.5	370.5; 811.0	
Serum viral load, number of copies/mL (range)*	0 (0–3,116.98)	0 (0–1,539.22)	0 (0–3,116.98)	0.658
Q1;Q3	0;0	0;0	0;0	
	n = 208	n = 140	n = 68	
Serum viral load in women, number copies/mL (range)**	0 (0–3,116.98)	0 (0–1,539.22)	0 (0–3,116.98)	0.725
Q1;Q3	0;0	0;0	0;0	
Viral load of genital secretions, number of copies/mL (range)**	0 (0–112,00)	0 (0–112,00)	0 (0–854,00)	0.043
Q1;Q3	0;0	0;0	0;0	

* P-value obtained through the Chi-square test with Yates correction for comparison of proportions. For comparison of medians the Mann Whitney U test was used.

** HIV positive partner in a couple.

The correlation between serum and genital viral load was further explored taking into account the presence of STIs which can exacerbate HIV genital shedding. There was a significant but weak correlation between genital secretion PCR results and serum virus load (r = 0.252; P<0.0001). Forty four of 208 HIV positive women (21.2%) had STIs that cause genital lesions. This included 26 cases of acquired syphilis (12.5%), 3 cases of herpes simples (1.4%), 17 cases of condyloma (8.2%), 2 cases of gonorrhea (1.0%), and 2 cases of candidiasis (1.0%). Genital secretion HIV-1 virus load was detectable in 4 of 44 women with STIs (9.1%), and in 12 of 164 subjects without STIs (7.3%) (P = 0.696). There was a significant correlation between PCR results of genital secretions and serum viral load in patients with STIs causing genital lesions (r = 0.359; P = 0.017). In the group without STIs, there was a significant but weaker correlation between genital and serum virus load (r = 0.219; P = 0.005).

Antiretroviral regimens used by subjects included multiple tablet regimens (MTR) in 177 participants (59%), and single tablet regimens (STR) for the remaining 41%. Index cases in stable relationships used STR more frequently than single individuals (45% vs. 33%; P = 0.047). Among 177 patients taking MTR, 146 were taking combination zidovudine/lamivudine plus a protease inhibitor (82.5%), 29 were on combination zidovudine/lamivudine, tenofovir disoproxil and a protease inhibitor (16.4%). Only 2 subjects (1.2%) were using more complex regimens which included an integrase inhibitor (raltegravir). The median time of ARV use was 4.2 years (range = 0.3 to 21.7 years), with no difference in median time of ARV use between index cases in partnerships and unpartnered individuals (4.5 years x 4.0 years; P = 0.307).

### Self-report of ARV adherence

As seen in Table 4, we noted a statistically significant association between self-reported adherence on ACASI and adherence identified through a biological marker, i.e., an undetectable serum viral load. Among 296 individuals who responded the ACASI question on adherence, 265 reported good adherence (89.5%). Serum virus load, the biological marker of adherence was undetectable in 230 of 296 participants (77.7%). There was a statistically significant association between good adherence reported on ACASI and an undetectable virus load result, with a RR of 1.6 and p value of < 0.0001 as shown in Table 4. However, 16 subjects classified as having low adherence per self-report still maintained an undetectable virus load at the time the questionnaire was answered (51.6% of low adherence responders). The proportion of women with undetectable viral load in vaginal secretions among those who reported good adherence was similar to those reporting low adherence (91.7% X 96.2%; P = 0.699) (Table 4). There was no difference in self-reported adherence between index cases in partnerships and unpartnered individuals (88.4% vs. 91.8%; P = 0.328).

**Table 4:** ACASI adherence responses contrasted with viral load results in serum (n = 296) and in genital secretions (n = 207).

	Undetectable serum viral load (N = 230)	Detectable serum viral load (N = 66)	OR (95%CI)	p value*
**Adherence reported by ACASI (n = 296)**	**N° (%)**	**N° (%)**		
Good adherence (n = 265)	214 (80.8)	51 (19.2)	3.93 (1.68–9.08)	0.001
Poor adherence (n = 31)	16 (51.6)	15 (48.4)	-	
	Undetectable serum viral load(N = 230)	Detectable serum viral load (N = 66)	OR (95%CI)	p value*
**Adherence report by ACASI (n = 207)**	**N° (%)**	**N° (%)**		
Good adherence (n = 181)	166 (91.7)	15 (8.3)	0.44 (0.01–3.14)	0.699
Poor adherence (n = 26)	25 (96.2)	1 (3.8)	-	

* p-value obtained with Fisher Exact test.

We noted a higher median family income in subjects with ARV adherence in our bi-variate analysis (R$ 1,500 X R$ 1,000; P = 0.001) as well in subjects who had their own income (79.6% X 70.3%; P = 0.095). Subjects who used STR were more adherent than those using MTR (86.2% X 71.2%; P = 0.002). In subjects with adequate ARV adherence, the median time of ARV use was higher than in those with poor adherence (4.6 years X 3.8 years; P = 0.08). We noted a trend towards higher adherence in subjects over 30 years of age (P = 0.176), subjects who were male (P = 0.250), and subjects with a high school education or higher (P = 0.239) We did not observe an association between ARV adherence and drug use (78.4% x 77.2%;OR = 1.1;95%CI:0.5–2.5;P = 0.871), occasional alcohol use (78.9%x77.2%; OR = 1.5;95%CI:0.5–4.0;P = 0.475) or frequent alcohol use (70.0%x77.2%;OR = 1.6;95%CI:0.6–4.6;P = 0.384). Having children or not also did not impact ARV adherence n (82.5% X 76.1%; OR = 1.5;95% CI:0.7–3.1; P = 0.306). In the logistic regression analysis, STR ARV use was independently associated with adherence as defined by an undetectable serum viral load. The use of MTR doubled the chance of ARV adherence as compared to MTR (Table 5).

**Table 5:** Factors independently associated with ARV adherence by multivariable analysis (n = 299).

	Adherence ARV (n = 231)	Poor adherence TRV (n = 68)	OR (IC 95%)	OR Adj	IC 95%	P
Age						
30 years or more (n = 250)	197 (78.8)	53 (21.2)	1.6 (0.8–3.1)	1.4	0.7–2.8	0.405
< 30 years* (n = 50)	35/50 (70.0)	15 (30.0)				
Gender						
Male (n = 92)	75 (81.5)	17 (18.5)	1.4 (0.8–2.6)	1.1	0.6–2.2	0.744
Female* (n = 208)	157 (75.5)	51 (24.5)	-			
Education						
Secondary or higher (n = 133)	107 (80.5)	26 (19.5)	1.4 (0.8–2.4)	1.4	0.8–2.4	0.284
Primary (n = 166)	124 (74.7)	42 (25.3)	-			
Married or in stable union						
Yes (n = 200)	156 (78.0)	44 (22.0)	1.1 (0.6–1.9)	1.1	0.6–2.0	0.719
No* (n = 100)	76 (76,0)	24 (24,0)	-			
Personal Income						
Yes (n = 226)	180 (79.6)	46 (20.4)	1.7 (0.9–3.0)	1.3	0.7–2.5	0.359
No* (n = 74)	52 (70.3)	22 (29.7)	-			
ARV regimen						
STR (n = 123)	106 (86.2)	17 (13.8)	2.5 (1.4–4.6)	2.3	1.2–4.4	0.011
MTR* (n = 177)	126 (71.2)	51 (28.8)				
Median time of ARV use, years, (range), Q1;Q3 (n = 300)	4.6 (0.3–21.7) 2.0; 8.0	3.8 (0.3–19.2) 2.0; 6.4	-	1.0	1.0–1.1	0.322

*categorical variable;

Primary = elementary school; Secondary = high school; STR = single tablet regimen; MTR = multiple tablet regimen.

## Discussion

We studied a population of serodiscordant couples and HIV unpartnered individuals at a period of time when there is universal recommendations for ARV use. During the time our study was conducted pre-exposure prophylaxis to partners of HIV-infected individuals was not the standard of care. Our main goal was to determine factors associated with adherence and adequate virologic plasma and genital suppression, both key to avoidance of HIV sexual transmission in this setting. As genital virus load can be significantly influenced by concurrent STIs we also evaluated our population for co-infections. We noted the differences between infectious markers such as blood and genital secretion viral load and contrasted findings with self-reports of adherence obtained via ACASI, taking into consideration also the presence of concurrent STIs. Few studies have compared partnered and unpartnered HIV-positive individuals in terms of ARV adherence. In one specific study, the presence of a partner was a strong predictor of adherence[25]. In our study population however, we did not identify significant differences regarding adherence when comparing index cases in serodiscordant couples and unpartnered individuals.

In our study, a fraction of subjects with undetectable serum viremia reported little adherence to ARV use on ACASI. Studies have demonstrated that this specific population is likely to subsequently experience treatment failure and should be targeted in efforts to improve adherence[26]. It is also known that reducing virus load in genital secretions through the use of ARV reduces the risk of HIV-1 sexual transmission, however little is known on the efficacy of ARV use in decreasing genital tract virus load taking into account gender differences, presence of STIs and HIV-1 subtype[27]. There is evidence that the genital tract of women infected with HIV-1 subtype C would function as a persistent reservoir of viral replication even during the use of ARV, positively affecting the sexual and perinatal transmission of HIV[27, 28]. Subtype analysis was only available for 25% of the infected subjects, however subtype C was the most prevalent virus clade identified.

In our study we identified a significant correlation between detectable genital HIV-1 and serum viremia in the presence of an STI with genital lesions, which is in accordance with the medical literature[27–29]. The presence of concurrent STIs can increase HIV-1 in genital secretions and thus increase the risk of HIV-1 transmission. Our findings underscore the pressing need to promptly diagnose and treat STIs among HIV serodiscordant couples. Overall however, measuring HIV-1 in vaginal secretions by PCR in all female patients did not prove to be a good marker of adherence.

One important finding in our study was that individuals in stable partnerships used ARVs in the STR format more frequently than unpartnered subjects, which may mean better adherence to treatment without the need of a change to more complex regimens. This is in accordance with Brazilian guidelines, as at the time of data collection, the recommendation of the Ministry of Health for first line therapy was the use of a single tablet containing tenofovir, lamivudine and efavirenz[30]. We noted a strong association between STR and adherence in our study, regardless of demographic, behavioral and prior duration of ARV use. In fact, contrary to what we anticipated, none of the behavioral factors evaluated, including substance use, were associated with adherence either by self-report or biomarkers. One caveat is that individuals on multi tablet regimens could more likely have experienced prior virologic treatment failure and therefore be more prone to lower adherence and a detectable viral load subsequently, a reflection of pill burden burnout. There are, however, a number of independent studies which have also demonstrated an association between STR, adherence and improved patient outcomes, including sustained virologic suppression, longer time to treatment discontinuation, retention in care, and fewer hospitalizations[31–34]. Nevertheless, the cross-sectional nature of our study precludes us from inferring possible causality between specific parameters, and provides a one-time snapshot of adherence in individuals followed in our hospital based clinics.

Study limitations therefore include the cross-sectional design and the unavailability of molecular testing for gonococcus, chlamydia and HPV at our institution (as is the case in most public hospitals in Brazil) at the time of study conduct. Adherence was monitored by self-report which can often overestimate adherence; as we saw in our study, 19% of patients reporting good adherence had detectable serum HIV RNA levels. The minimal level of HIV genital RNA required for efficient transmission of HIV is presently unknown, which can affect interpretability of results. Nevertheless our findings can be used to plan further longitudinal assessments.

In summary, our results highlight critical issues pertaining to ARV adherence and virologic suppression in HIV infected partnered and unpartnered individuals, with the take home message that single tablet ARV regimens may be the most important tool in fostering adherence in our population, regardless of social, behavioral or demographic parameters. It is important to note that even though serum virus load is known to be highly predictive of HIV transmission in multiple studies, in our population we did find discrepant results between serum and genital secretions in a small proportion of women evaluated. In light of this finding, we cannot assume that there is zero risk of transmission in all HIV-infected individuals with serologic suppression.

## Supporting information

S1 Dataset Tripai dataset used in analysis. Identifying information redacted.
(XLSX)
